# Physical inactivity and sitting time prevalence and trends in Mexican adults. Results from three national surveys

**DOI:** 10.1371/journal.pone.0253137

**Published:** 2021-07-02

**Authors:** Catalina Medina, Alejandra Jáuregui, Cesar Hernández, Teresa Shamah, Simón Barquera

**Affiliations:** 1 Center for Health and Nutrition Research, National Institute of Public Health, Cuernavaca, Morelos, Mexico; 2 Center for Evaluation and Surveys Research, National Institute of Public Health, Cuernavaca, Mexico; Universitat de les Illes Balears, SPAIN

## Abstract

**Background:**

Physical inactivity and high sitting time are directly related to mortality and morbidity of non-communicable diseases (NCDs). Thus, improved understanding of the prevalence and trends of these behaviors could support the design of policies and interventions for NCDs prevention.

**Objective:**

To determine the current prevalence of physical inactivity and high sitting time, to analyze the trends, and to estimate the association of meeting/not meeting physical activity recommendations and low/high sitting time with sociodemographic characteristics and body mass index categories.

**Methodology:**

Data from the 2018 National Health and Nutrition Survey were used. Moderate-to-vigorous physical activity (MVPA) and sitting minutes per week were calculated using the International Physical Activity Questionnaire short form (IPAQ). In total, 38,033 questionnaires of adults aged 20 to 69-year-old were analyzed. Adults were classified as physically inactive if they achieved less than 150 minutes per week of MVPA and as with high sitting time if they accumulated more than 420 minutes of sitting per day. Health and Nutrition National Surveys (ENSANUT) 2006, 2012 and 2018 were used to estimate the trends.

**Results:**

In total, 16.5% were classified as physically inactive and 11.3% within the high sitting time category. Both prevalences increased more than 40% during the 12-y period (2006–2018). In 2018, men, younger adults, those living in urban areas, and people within the highest socioeconomical status and educational levels were more likely to not achieve physical activity recommendations and to be classified in the highest sitting time category.

**Conclusion:**

To stop current increased trends and achieve global targets, stronger and more concerted efforts to promote physical activity and reduce sitting time are required. Thus, continued surveillance of these behaviors is necessary.

## Introduction

Physical inactivity is associated with increased morbidity and mortality of many diseases including diabetes, hypertension, cancer, stroke and cardiovascular diseases [1, 2]. In addition, physical inactivity is responsible for a substantial economic burden [3]. Based on the World Health Organization (WHO) recommendations, physical inactivity is defined as failing to accumulate at least 150 minutes of moderate physical activity or 75 minutes of vigorous physical activity or the combination of both intensities per week [4]. Globally, 27.5% of adults are classified as physically inactive [5]. Physical inactivity prevalence has not experienced a significant increase worldwide since 2001 [5]. However, a significant change of 44% was observed in Mexico between 2006 to 2012 [6].

Physical inactivity and sedentary behaviors are two distinct behaviors [7]. Sedentary behaviors have been defined as those activities whose energy expenditure is ≤1.5 metabolic equivalent (METs), while in seated, reclined or lying posture [8]. Sitting time is the most common sedentary behavior. Several studies have described that prolonged time in sedentary behaviors might increase the incidence and mortality of many chronic diseases, independent of total reported physical activity [7, 9–14]. The available evidence suggests that total sitting time per day has increased in the last years [15]. Furthermore, prolonged sitting time has been observed in men, people within the higher income level [15], and those in the highest BMI category [15]. Although, there is not a global cut-off point to estimate the prevalence of high sitting time in adults, most of the recently published studies have used the highest quartile ranging from 7 to more than 8 hours per day [16–18].

Physical inactivity and high sitting time prevalence, as well as trends have been monitored through the national surveillance system in Mexico since 2006 [6, 17], however there is a lack of information related to the current prevalence stratified by sociodemographic and biological indicators. Thus, the purposes of this study were to determine the prevalence of physical inactivity and high sitting time, to analyze the trends, and to estimate the association between meeting/not meeting physical activity recommendations and being in the low/high sitting time category and sociodemographic characteristics and body mass index categories.

## Methodology

### Health and Nutrition Surveys and participants

We used information from the 2018 Health and Nutrition National Survey (by its acronym in Spanish: ENSANUT). This survey used a probabilistic multistage stratified cluster sampling design and is representative of Mexican adult population. ENSANUT 2018 was conducted between June and February 2018–2019 [19]. Data of 38,935 adults aged 20–69 years old was collected. For this analysis, data from 38,033 adults was analyzed after excluding 0.5% pregnant women, 1.3% with missing physical activity information and 0.5% with invalid sitting time information (n = 902). ENSANUT 2006 and 2012 have been previously described [20, 21]. Both surveys were cleaned and analyzed as 2018. All participants provided written informed consent. The Ethics Review Board from the National Institute of Public Health reviewed and approved this study. Detailed description of survey’s methodology has been described elsewhere [19–21].

### Physical activity and sitting time assessment and data cleaning

The Spanish version of the International Physical Activity Questionnaire (IPAQ) short form was used to measure physical activity levels and sitting time among ENSANUTs [6]. This questionnaire has been validated in Mexican adults aged 20–69 years old [22]. It was applied by trained interviewers using a computer in 2018. The IPAQ short form asks for minutes and days of vigorous, and moderate physical activity, as well as walking, accumulated in at least 10 consecutive minutes over the last 7 days [23]. In addition, this questionnaire asks for sitting hours per day on a weekday or on Wednesday.

Data cleaning was performed using the IPAQs protocol [24]. Minutes per week of vigorous (VPA) and moderate (including walking) physical activity (MWPA) were calculated for each participant. Minutes per week of moderate-to-vigorous physical activity (MVPA) were estimated by adding moderate and vigorous physical activities per week. To calculate sitting time, sitting minutes per day <10 minutes and >16 hours were eliminated from the analysis [24].

### Physical inactivity (uncorrected and corrected for overestimation) and sitting time prevalences

Physical inactivity prevalence was classified as uncorrected and corrected for overestimation. For uncorrected physical activity prevalence, minutes per week of VPA were multiplied by two and added to the minutes per week of MWPA resulting in MV2PA minutes per week. This variable was used to classified adults as inactive (<150 minutes per week), moderately active (150–299 minutes per week) and sufficiently active (≥300 minutes per week) based on the WHO physical activity recommendations [4]. Then, physical activity was categorized as 1) inactive and 2) active (moderately and sufficiently active). Due to the already known overestimation of physical activity using self-reports [22], corrected prevalence was estimated using a previously developed equation (corrected prevalence) [6]. This equation was applied to the MV2PA minutes per week and stratified using the abovementioned WHO physical activity recommendations.

Currently there is not an available cut-off point to estimate the sitting time prevalence. However, based on previous studies we categorized minutes per day of sitting as sedentary (>420 minutes per day) and non-sedentary (≤420 minutes per day) [17].

### Categories for being classified as inactive/active or sedentary/non-sedentary

Three groups were created combining physical activity and sitting time categories: 1) active and non-sedentary, and 2) inactive and non-sedentary or active and sedentary, and 3) inactive and sedentary.

### Sociodemographic and anthropometric variables

Several sociodemographic (sex, age, area, socioeconomic status, and educational levels) and anthropometric (body mass index) variables were asked or measured. Participants were classified based on whether they lived in a rural (<2,500 residents) or in an urban area (≥2,500 residents). Household socioeconomic status was measured using a wellness condition index based on eight key components that assessed household characteristics, services and belongings: construction materials of the floor, ceiling and walls, water accessibility, vehicle ownership, sleeping rooms, household belongings (refrigerator, washing machine, microwave, stove, boiler), electrical goods (computer, telephone, television, radio) [25]. This index was categorized in tertiles: low, medium and high.

Weight and height were measured by trained personnel. Weight was assessed to the nearest 0.1 kg; height was measured to the nearest 0.1 cm. BMI was calculated and stratified based on WHO adult cut-off points: underweight (<18.5 kg/m^2^), normal weight (18.5–24.9 kg/m^2^), overweight (25–29.9 kg/m^2^) and obese (≥30 kg/m^2^) [26].

### Statistical analysis

Percentages and confidence intervals were used to describe the sample. Mean minutes per week of MVPA and sitting time were logarithmically transformed. To assess the trends among years, uncorrected physical inactivity and sitting time prevalence was age-standardized to reflect the average age structure of the world’s population using WHO distribution as a reference [27]. Physical inactivity prevalence was compared to previously reported prevalences (2006 and 2012) [6]. In addition, general linear models were used to estimate differences in the mean minutes per week of MVPA and sitting time and prevalence by survey. Kernel-density plots were used to project the proportional amount and the distribution of mean MVPA and sitting time per week by survey (2006, 2012, 2018).

To estimate the association between active/inactive and sedentary/non-sedentary, with sociodemographic characteristics and BMI categories, we used a multinomial logistic regression test for the 2018 survey. We considered active and non-sedentary as a reference group. Multivariate model was adjusted by sex, age, area, household socioeconomic status and education level. Models were tested for multicollinearity. Significance was set at p<0.05. In order to maintain the representative nature of the sampling design, weights were considered using complex samples analysis in SPSS software version 25 (IBM SPSS statistics, IBM Corporation, Somers, NY).

## Results

Table 1 shows the demographic characteristics and BMI of the participants. Above 10% individuals were adults aged 60-69-year. The prevalence of obesity was 37.0%. In total, more than 20% of adults lived in rural areas. A lower number of participants were classified into the less than primary education category.

**Table 1:** Sociodemographic and anthropometric characteristics. ENSANUT 2018.

Variables	n	% (95% CI)
**Gender**		
Men	17,174	45.3 (44.6, 46.1)
Women	20,859	54.7 (53.9, 55.4)
**Age group**		
20–29	8,561	25.6 (24.9, 26.3)
30–39	9,508	23.1 (22.4, 23.7)
40–49	8,712	22.0 (21.3, 22.6)
50–59	6,445	17.4 (16.8, 17.9)
60–69	4,807	12.0 (11.5, 12.6)
BMI classification*		
Underweight	138	1.2 (0.9, 1.5)
Normal weight	3,145	22.6 (21.5, 23.7)
Overweight	5,636	39.2 (38.0, 40.4)
Obesity	5,510	37.0 (35.8, 38.3)
**Areas**		
Rural	10,025	20.8 (20.3, 21.3)
Urban	28,008	79.2 (78.7, 79.7)
**Socioeconomic status**		
Low	12,380	28.4 (27.7, 29.2)
Medium	12,780	32.2 (31.4, 33.0)
High	12,871	39.4 (38.5, 40.2)
**Educational levels**		
Less than primary	1,546	3.8 (3.6, 4.2)
Primary or secondary	20,616	51.5 (50.6, 52.3)
High school or higher	15,871	44.7 (43.9, 45.5)

*BMI is available only for n = 14,429.

Table 2 describes the uncorrected and corrected physical activity prevalence and sitting time prevalence. Based on the uncorrected physical inactivity prevalence, an increment of 43.5% was observed from 2006 (11.5%) to 2018 (16.5%) (p<0.05). The same pattern was observed for the corrected physical inactivity prevalence in the same period of time (14% vs. 20.8%). With respect to high sitting time prevalence, a statistically significant increase was observed between 2006 (7.6%) and 2012 (9.5%) (25% increase), 2012 and 2018 (11.3%) (19% increase), and 2006 and 2018 (49% increase).

**Table 2:** Prevalence and trends of physical activity and sitting time. ENSANUT 2006, 2012, 2018.

Variables	2006^a^ % (95%CI)	2012^b^ % (95%CI)	2018^c^ % (95%CI)
**Physical activity prevalence (uncorrected)**			
Inactive	11.5 (11.1, 12.3)^b,c^	15.4 (14.3, 16.7)	16.5 (16.0, 17.1)
Moderately active	6.8 (6.6, 7.2)^b,c^	11.1 (10.0, 12.3)	11.4 (10.9, 11.9)
Sufficiently active	81.6 (80.8, 82.3)^b,c^	73.5 (72.1, 75.0)	72.1 (71.4, 72.2)
**Physical activity prevalence (corrected)**			
Inactive	14.0 (13.4, 14.7)^b,c^	19.2 (17.9, 20.6)^c^	20.8 (20.1, 21.5)
Moderately active	20.3 (19.6, 21.0)^b,c^	29.4 (27.8, 31.1)	30.9 (30.2, 31.6)
Sufficiently active	65.7 (64.8, 66.6)^b,c^	51.4 (49.6, 53.2)^c^	48.3 (47.4, 49.1)
**Sitting time prevalence**			
Low	92.4 (91.8, 92.7)^b,c^	90.5 (89.4, 91.6)^c^	88.7 (88.1, 89.2)
High	7.6 (7.2, 8.1)^b,c^	9.5 (8.4, 10.6)^c^	11.3 (10.8, 11.8)

Uncorrected for self-report overestimation.

Corrected for self-report overestimation.

^a,b,^ statistically significant differences between years

Uncorrected physical activity prevalence and sitting prevalence were age-standardized.

**Fig 1:**
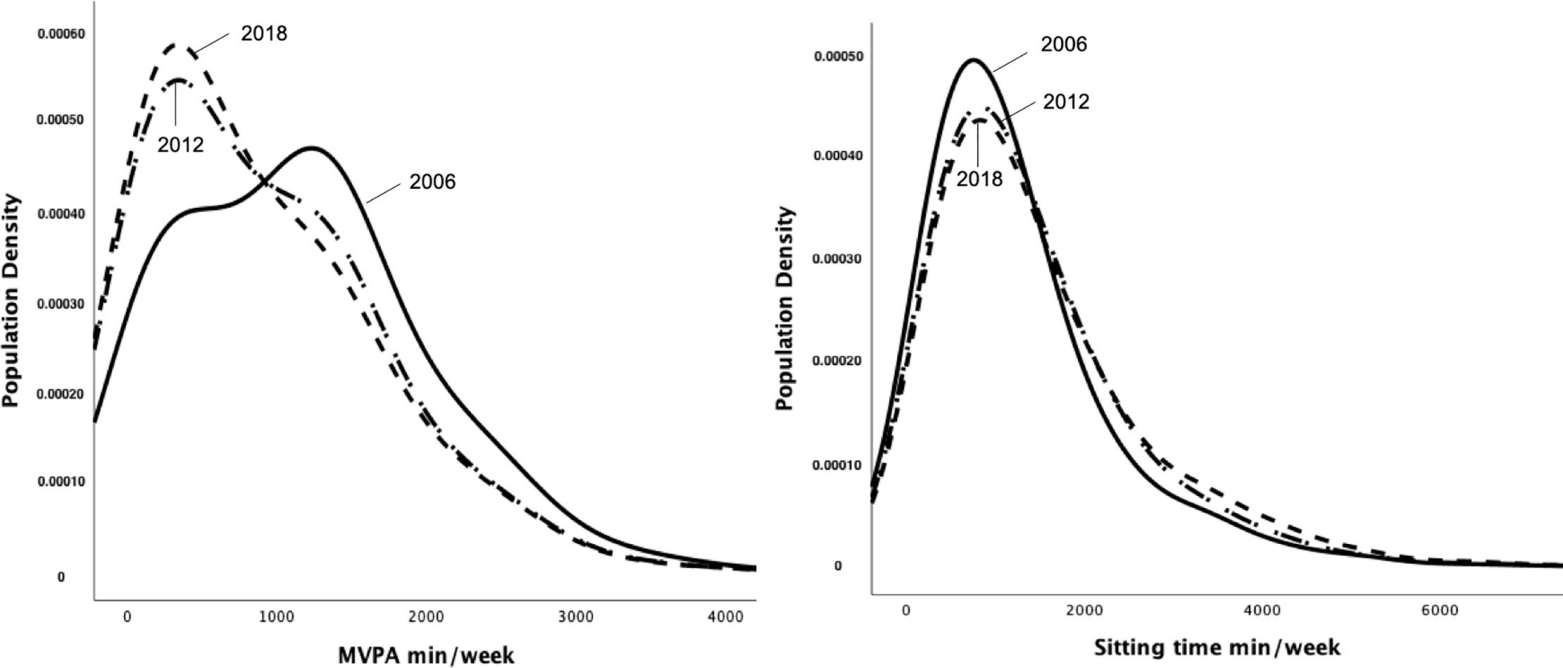
Mean minutes per week of moderate-to-vigorous physical activity and sitting time stratified by survey. a and b.

As shown in Fig 1A, the distribution of minutes per week of physical activity shifted slightly to the left, whereas the opposite was observed for the distribution of minutes per week of sitting time (Fig 1B). This means that the average minutes per week of physical activity has decreased, and the average minutes per week of sitting time has increased. In addition, the mean minutes per week of MVPA was significantly higher in 2006 compared to minutes reported in 2012 and 2018 (1104.5 minutes vs. 872.7 and 814.9 minutes, respectively) (p<0.01). Mean weekly minutes of sitting time was significantly lower in 2006 with respect to minutes per week in 2012 and 2018 (1284.9 minutes vs. 1454.7 and 1503.2 minutes, respectively) (p<0.01).

According to Table 3, statistically significant differences were observed in minutes per week of MVPA and sitting minutes per week between groups. Statistically significant differences were observed on MVPA minutes per week for men vs. women, those aged 50–69 years vs. other age groups, underweight and obese groups (vs. other groups, respectively) and those living in a rural vs. urban areas, and those in the lowest socioeconomic status and educational levels.

**Table 3:** Differences in mean for moderate-to-vigorous physical activity and sitting time minutes per week as measured by ENSANUT 2018.

Variables	Moderate-to-vigorous physical activity minutes per week	Sitting time minutes per week
	Mean (95%CI)	Mean (95%CI)
**Gender**		
Men ^1^	920.2 (902.1, 938.2)^2^	1629.4 (1600.6, 1658.2)^2^
Women ^2^	727.7 (713.7, 741.6)	1398.6 (1376.1, 1421.2)
**Age group**		
20–29 ^1^	830.7 (808.8, 852.5)^4,5^	1681.1 (1642.1, 1720.1)^2,3,4,5^
30–39 ^2^	845.1 (823.9, 866.3)^4,5^	1511.2 (1473.3, 1549.2)^3,4^
40–49 ^3^	854.2 (831.1, 877.2)^4,5^	1428.2 (1392.9, 1463.5)
50–59 ^4^	796.9 (770.8, 822.9)	1377.7 (1336.7, 1418.7)
60–69 ^5^	678.2 (649.9, 706.7)	1427.7 (1390.3, 1475.0)
BMI classification*		
Underweight ^1^	663.2 (502.1, 824.4)^2,3,4^	1324.0 (1042.9, 1605.0)
Normal weight ^2^	878.2 (841.1, 915.2)^4^	1399.2 (1335.1, 1463.2)
Overweight ^3^	846.2 (816.0, 876.4)^4^	1366.0 (1322.8, 1409.2)
Obesity ^4^	760.8 (734.2, 787.5)	1420.0 (1375.9, 1464.2)
**Areas**		
Rural ^1^	923.9 (898.8, 948.9)^2^	1117.7 (1086.9, 1143.4)^2^
Urban ^2^	786.3 (772.9, 799.8)	1604.5 (1582.6, 1626.)
**Socioeconomic status**		
Low ^1^	904.3 (882.9, 925.7)^2,3^	1168.9 (1141.8, 1196.1)^2,3^
Medium ^2^	821.2 (801.1, 841.3)^3^	1478.9 (1448.6, 1509.3)^3^
High ^3^	743.8 (727.0, 760.6)	1764.5 (1730.9, 1797.9)
**Educational levels**		
Less than primary ^1^	815.6 (760.9, 870.2)^2,3^	1133.7 (1047.1, 1220.3)^2,3^
Primary or secondary ^2^	859.3 (842.2, 876.5)	1268.5 (1246.1, 1290.9)^3^
High school or higher ^3^	763.8 (748.3, 779.2)	1805.3 (1775.2, 1835.4)

^1,2,3,4,5^ statistically significant differences within categories of sociodemographic variables.

*BMI is available only for n = 14,429.

With respect to sitting time, significant differences were observed in sitting minutes per week between men vs. women, those aged 20–29 years vs. other age groups, rural vs. urban areas, low and medium socioeconomic status vs. high socioeconomic status, and less than primary and primary or secondary vs. high school or higher.

In 2018, after controlling for confounders, women (vs. men), those adults aged >49 years old (vs. other age groups), adults classified as underweight, those within the highest socioeconomic status (vs. the lowest) and adults classified in the primary educational level (vs. others) were more likely to be classified as active/inactive or sedentary/non-sedentary.

Men (vs. women), adults <40 years (vs. other age groups), those living in urban areas, individuals classified within the highest socioeconomic status (vs. lower) and those in the high school or higher group (vs. other educational level groups) were more likely to be classified as inactive and sedentary (Table 4).

**Table 4:** Multinomial regression analysis for categories of physical activity and sitting time by sociodemographics and BMI. ENSANUT 2018.

	Inactive and non-sedentary or Active and sedentary		Inactive and sedentary	
Variables (OR (95% CI) n = 38,033	Univariate Model	Multivariate Model	Univariate Model	Multivariate Model
**Gender**				
Women	1	1	1	1
Men	**0.73 (0.67, 0.79)**	**0.74 (0.68, 0.80)**	**1.37 (1.20, 1.53)**	**1.33 (1.18, 1.50)**
**Age group**				
20–29	1	1	1	1
30–39	1.04 (0.92, 1.19)	1.01 (0.89, 1.16)	**0.78 (0.67, 0.92)**	0.86 (0.73, 1.01)
40–49	**1.14 (1.00, 1.30)**	1.09 (0.96, 1.24)	**0.63 (0.53, 0.75)**	**0.72 (0.60, 0.85)**
50–59	**1.29 (1.14, 1.47)**	**1.22 (1.07, 1.40)**	**0.47 (0.38, 0.58)**	**0.52 (0.42, 0.65)**
60–69	**1.97 (1.71, 2.27)**	**1.82 (1.57, 2.11)**	**0.45 (0.35, 0.57)**	**0.57 (0.44, 0.74)**
BMI classification*				
Normal weight	1	1	1	1
Underweight	**1.93 (1.02, 3.64)**	**1.98 (1.05, 3.73)**	0.93 (0.41, 2.07)	0.75 (0.33, 1.69)
Overweight	0.99 (0.83, 1.19)	0.93 (0.77, 1.12)	0.88 (0.67, 1.17)	0.94 (0.71, 1.24)
Obesity	**1.34 (1.11, 1.62)**	1.17 (0.98, 1.41)	0.87 (0.67, 1.14)	1.00 (0.77, 1.33)
**Areas**				
Rural	1	1	1	1
Urban	**1.15 (1.06, 1.26)**	1.09 (0.99, 1.20)	**3.19 (2.69, 3.78)**	**1.94 (1.61, 2.33)**
**Socioeconomic status**				
Low	1	1	1	1
Medium	**1.15 (1.05, 1.27)**	**1.16 (1.04, 1.28)**	**2.26 (1.90, 2.68)**	**1.64 (1.37, 1.96)**
High	**1.24 (1.13, 1.37)**	**1.25 (1.11, 1.40)**	**3.66 (3.10, 4.30)**	**2.16 (1.80, 2.59)**
**Educational levels**				
Primary or less	1	1	1	1
Secondary	**0.73 (0.59, 0.89)**	**0.80 (0.66, 0.98)**	**2.31 (1.29, 4.11)**	1.52 (0.85, 2.71)
High school or higher	**0.67 (0.55, 0.82)**	**0.74 (0.60, 0.91)**	**6.91 (3.89, 12.2)**	**3.02 (1.68, 5.45)**

∞Active and non-sedentary as reference group.

*BMI classification was based on a subsample (n = 14,429).

Multivariate Model–adjusted for sex, age, area, socioeconomic status and education level.

## Discussion

The purposes of this study were to determine the current prevalence of physical inactivity and sitting time, to analyze the trends, and to estimate the association between meeting/not meeting physical activity recommendations and having a low/high sitting time, with sociodemographic characteristics and body mass index categories in Mexico. Main results indicated that uncorrected physical inactivity prevalence was 16.5%, this increased 43.5% from 2006 to 2018, the same pattern was observed for the corrected prevalence (14% to 20.8%). In the same way, sitting time prevalence increased 49% between 2006 and 2018. In addition, based on the multivariate model, men, younger adults, those living in urban areas, those within highest socioeconomic status and those within the highest educational level were more likely to not meet physical activity recommendations and have high sitting time.

We observed an increment in the uncorrected and corrected physical inactivity prevalence in the last 12 years. This result is in line with a global study indicating that although the global physical inactivity prevalence did not change substantially between 2001 and 2016 (28.5% vs. 27.5%) [5] a significant increase was observed for the Caribbean and Latin American regions [5]. Specifically, this study reported a corrected physical inactivity prevalence of 28.9% in Mexico in 2016 compared to the 20.8% reported in this study in 2018. Among the reasons for this difference could be the distinct constants used within the equations (i.e., a global constant was used for all regions while this study used one derived from a study among Mexican adults), and questionnaire adjustments due to the use of different international self-reports (example: GPAQ vs. IPAQ) [5, 22].

According to mean minutes per week of MVPA women vs. men, those aged 60–69 years old vs. other age groups, and those classified as underweight and obese were the most inactive population groups. This result is similar to previous international [5, 28] and national studies [6, 22, 29, 30]. This could be explained by the fact that women and older adults [31] are involved in light intensity physical activity mainly performed within leisure or home-related activities [32] and adults classified as underweight or obese could have decreased levels of physical activity and an increased sedentarism [33, 34]. In addition, the lack of frequently use of places for physical activity by Mexican women could be another reason [29].

Even though the sitting time prevalence has increased in the last 12 years in Mexico, this prevalence is lower than that reported in other countries [16]. For instance, data from the US reported that the prevalence of high sitting time was 25.7% in adults [16] and 18.5% in the European Union [18] compared to the 11.3% reported in this study. Discrepancies could be related to the high sitting time cut-off point [16, 18]. Until now there is not an official cut-off point for sedentary behaviors, however, some authors have reported that spending 7–8 hours per day sitting could be detrimental for health [17, 35].

A study reporting the median hours per day of sitting time indicated that people from upper-middle income countries spent on average 234 minutes per day [36]. This result was slightly higher compared to the one obtained in our study (214.7 minutes per day) [36]. In addition, men, younger adults, those living in urban areas, those within the highest SES and educational levels spent a higher number of minutes per week on sitting time compared to other groups. This could be related to the fact that people in these groups spent more time in sedentary occupations such as industries, office works and schools.

Men, younger adults, those living in urban areas, people within the medium and high SES and within the highest educational levels were more likely to not meet physical activity recommendations and have high sitting time. Similar results have been observed in a previous study that estimated the association of high sitting (≥7.5 hour per day)/less active physical activity (0–5 days) using the IPAQ [37]. However, some differences have been reported in a European study that used accelerometers and higher sitting time cut-off points (>10 hours per day) [38]. As shown in previous studies [37], similar groups with higher ORs of being high sitters [17] are also at risk of being classified as sedentary/non-active. Thus, caution should be taken in these groups because previous evidence has shown that high sitting time (≥4 h/d) is associated with all-cause [39, 40] and cardiovascular mortality [39] in the least active group (<150 minutes/week).

Although several strategies to increase physical activity and decrease sitting time have been taking place in Mexico [41–43], such as “chécate, mídete [41], muévete”, and “pausas por la salud” [42], these are limited to informational approaches [41], their reach is limited [42] and evidence indicates lack of clarity within the implementation [43]. Hence, the prevalence of both behaviors is still increasing. This trend is opposite to the WHO’s global target for a reduction of 10% in physical inactivity by 2025 and of 15% by 2030 [44]. A Mexican study estimated that a 15% reduction in physical inactivity could decrease more than 1,500 cases of type 2 diabetes and cardiovascular disease [2]. Thus, focalized strategies that could promote physical activity levels and reduce sitting time should be implemented.

Nowadays, the COVID-19 pandemic has changed the daily life worldwide. These changes have impacted the physical activity and sitting time levels among the population and in the Mexican population [45]. Although the WHO has recommended several strategies for creating active places and people [44], many opportunities to be physically active have been suspended. The COVID-19 era [46] has brought the use of webpages and free of charge applications to increase physical activity levels at home [47]. However, this will not be enough to reduce physical inactivity and sedentary levels in the population. Therefore, it is necessary to identify strategies that could help to improve active lifestyles considering the new social norms.

Several limitations should be considered in the interpretation of our results. Physical activity and sitting time were measured using IPAQ short, not allowing to differentiate physical activity in the four domains in which it is performed (occupational, house, commuting and leisure time) and limiting the ability to inform domain-specific physical activity promotion policies and strategies. Self-reported measures could over and underestimate, respectively. Adjustment of physical activity levels with an equation developed for Mexican population may attenuate the overestimation of physical activity [6]. However, there is no similar procedure to adjust IPAQ self-reported sitting time estimates. Due to study design, causal association between physical activity and sociodemographic characteristics cannot be inferred.

## Conclusion

Contrary to global targets for reducing physical inactivity by 2030, the prevalence of physical inactivity and high sitting time have increased in the last 12 years in Mexico among adults. Such trends indicate that current national programs and policies are failing to promote physical activity and to reduce sitting time. To halt current trends and achieve global targets, stronger and more concerted efforts to promote healthy behaviors are required. These results encourage the continued monitoring of physical activity and sedentary time through national surveys.
